# Writing motivation and ability profiles and transition during a technology-based writing intervention

**DOI:** 10.3389/fpsyg.2023.1196274

**Published:** 2023-06-21

**Authors:** Tania Cruz Cordero, Joshua Wilson, Matthew C. Myers, Corey Palermo, Halley Eacker, Andrew Potter, Jessica Coles

**Affiliations:** ^1^School of Education, University of Delaware, Newark, DE, United States; ^2^Measurement Incorporated, Durham, NC, United States; ^3^Arizona State University, Tempe, AZ, United States

**Keywords:** motivation, self-efficacy, writing, middle school, automated writing evaluation (AWE), latent profile analysis

## Abstract

Students exhibit heterogeneity in writing motivation and ability. Profiles based on measures of motivation and ability might help to describe this heterogeneity and better understand the effects of interventions aimed at improving students’ writing outcomes. We aimed to identify writing motivation and ability profiles in U.S. middle-school students participating in an automated writing evaluation (AWE) intervention using MI Write, and to identify transition paths between profiles as a result of the intervention. We identified profiles and transition paths of 2,487 students using latent profile and latent transition analysis. Four motivation and ability profiles emerged from a latent transition analysis with self-reported writing self-efficacy, attitudes toward writing, and a measure of writing writing: Low, Low/Mid, Mid/High, and High. Most students started the school year in the Low/Mid (38%) and Mid/High (30%) profiles. Only 11% of students started the school year in the High profile. Between 50 and 70% of students maintained the same profile in the Spring. Approximately 30% of students were likely to move one profile higher in the Spring. Fewer than 1% of students exhibited steeper transitions (e.g., from High to Low profile). Random assignment to treatment did not significantly influence transition paths. Likewise, gender, being a member of a priority population, or receiving special education services did not significantly influence transition paths. Results provide a promising profiling strategy focused on students’ attitudes, motivations, and ability and show students’ likeliness to belong to each profile based on their demographic characteristics. Finally, despite previous research indicating positive effects of AWE on writing motivation, results indicate that simply providing access to AWE in schools serving priority populations is insufficient to produce meaningful changes in students’ writing motivation profiles or writing outcomes. Therefore, interventions targeting writing motivation, in conjunction with AWE, could improve results.

## 1. Introduction

Writing is a key skill for academic success but results from national tests paint a discouraging picture about U.S. middle-schoolers’ writing performance. In the last National Assessment of Educational Progress (NAEP) writing assessment in 2011, proficiency rates for writing were alarmingly low; only 27% of eighth graders performed at or above the proficient level. Rates were lower for Black and Hispanic/Latinx students (only 10 and 13% at or above proficient, respectively), and students from low-income households as indicated by their receiving free/reduced-priced lunch (FRL; only 12% at or above proficient) (National Center for Education Statistics, 2012). In addition, boys have historically underperformed girls in writing (Reilly et al., 2019), and the intersection of gender, race, and socio-economic status may exacerbate or ameliorate relative risk of writing difficulty. Given this scenario, intervention is urgent, especially for these populations.

A potential avenue for intervention is to improve students’ motivation and attitudes toward writing as these characteristics are essential for writing development given the significant cognitive and motivational resources required to initiate, sustain, and monitor writing behaviors (Deane, 2018; Graham, 2018). Further, motivation and attitudes toward writing are highly predictive of writing ability (Graham et al., 2018). Previous studies have emphasized that measures of motivation and attitudes toward writing can be used to identify struggling writers (Coker et al., 2018), and can be impacted by interventions to improve writing ability. For example, technology-based writing interventions can support writing instruction and positively impact writing ability (Ekholm et al., 2018) and motivation (Morphy and Graham, 2012). A meta-analysis found that word processing had large, positive effects on struggling writers’ motivation to write (ES = 1.42) and moderate effects on writing ability (ES = 0.52) (Morphy and Graham, 2012). One promising technology-based writing intervention is *automated writing evaluation* (AWE). AWE is software that provides immediate, automated feedback, often in conjunction with evaluative scores (Hockly, 2019; Strobl et al., 2019; Deeva et al., 2021). AWE has shown promise for increasing students’ persistence at solving problems in their writing (Wilson and Czik, 2016), their motivation to revise (Moore and MacArthur, 2016), and their writing self-efficacy (Wilson and Roscoe, 2020).

However, this begs the question of how best to characterize students’ motivation and attitudes toward writing (Camacho et al., 2021a). This is especially important in middle school when students’ motivation and attitudes toward writing significantly worsen (Wright et al., 2020). Moreover, it is important to understand what motivation and writing ability look like for students more likely to struggle with writing (i.e., Black and/or Hispanic students who receive FRL). Furthermore, research is needed to help understand how motivational profiles may change over time and in response to intervention, particularly promising technology-based interventions such as AWE.

### 1.1. Theoretical framework

#### 1.1.1. Writing motivation constructs

Writing motivation is a complex umbrella for numerous constructs and definitions. Overall, it refers to the “orientation to writing that is triggered, stimulated, and to some degree manipulated by the attractive and challenging features of the activity that emerge in a specific situation” (Boscolo et al., 2012, p. 31). There have been multiple approaches to parse the components of writing motivation and there is an ongoing debate on how to conceptualize it and assess it (see Abdel Latif, 2019 for a discussion). Accounts such as Graham’s (2018) and Graham et al. (2022) define writing motivation as a multidimensional construct, comprised by a set of different beliefs: about identity as a writer, reasons for writing, the value of writing, writing goals, interests and attitudes toward writing, competence as a writer, reasons for succeeding in writing, and beliefs about the community setting in which one writes.

Empirical studies have aimed to untangle the constructs under writing motivation. A recent systematic review on the topic found at least 24 different constructs that have been measured as writing motivation in the past decades (Camacho et al., 2021a). The most well-researched constructs were self-efficacy for writing and writing attitudes (Abdel Latif, 2019; Camacho et al., 2021a). These constructs had strong, positive relationships with writing ability. Specifically, self-efficacy had the strongest relation (*r* = 0.60) but writing attitudes (*r* = 0.15–0.34), and enjoyment of writing (*r* = 0.32) had positive associations too (Camacho et al., 2021a).

Self-efficacy refers to the judgment of one’s ability to conduct a task and is often a “cognitive mediator” for actions (Bandura, 1982, p. 126). Applied to writing, self-efficacy refers to the confidence one has to complete certain writing tasks successfully (Bruning et al., 2013). Writing self-efficacy has been the most researched construct in the realm of writing motivation (Camacho et al., 2021a), and as such, there are multiple conceptualizations and assessments (Abdel Latif, 2019).

Bruning et al. (2013) critiqued early accounts of self-efficacy because they measured the trait globally, based only on writing activities and outcomes. This approach ignores the psychological and linguistic features of writing that can impact the definition of self-efficacy. Hence, Bruning et al.’s (2013) model of writing self-efficacy accounts for these multiple underlying factors and proposes three dimensions of self-efficacy. First, *conventions* refer to the confidence in the writer’s ability to comply with generally accepted writing standards in a language while putting ideas into writing. This dimension includes, for example, spelling, morphology, sentence combining, etc. Second, *idea generation* refers to the confidence in the writer’s ability to generate ideas while writing, and the ability to correctly connect them. This dimension accounts for the cognitive processes involved in writing and is closely related to semantics and schematics. Finally, *self-regulation* refers to the confidence in the writer’s ability to successfully navigate the many dimensions, subtasks, and barriers in the writing process. This dimension relates to the management, monitoring and evaluation of writing. These three dimensions of self-efficacy in writing were moderately and positively related to attitudes about writing (*r* = 0.22–0.50) and writing ability (*r* = 0.20–0.38; Bruning et al., 2013).

Attitudes about writing refer to positive or negative affect toward writing or aspects of it (Graham, 2018; Camacho et al., 2021a). Positive attitudes toward writing have been directly associated with improved writing ability and this construct has been deemed more malleable than other motivational constructs (Graham et al., 2007). Writing attitudes tend to decline over the years (Ekholm et al., 2018). Furthermore, attitudes toward writing, measured as how much one likes or dislikes writing, have been shown as an independent motivation construct related to both self-efficacy and writing ability (*r* = 0.13, Bruning et al., 2013; MacArthur et al., 2016).

Writing self-efficacy and attitudes toward writing are well-defined, independent constructs under writing motivation, and the relations between them and with writing ability has been largely established (Abdel Latif, 2019). Therefore, profiling with measures of these constructs along with a measure of writing ability can allow us to explore in more detail the relations among them, how these constructs interact in a priority population, and how responsive they are to a writing intervention.

#### 1.1.2. Profiles of students’ motivation and ability in relation to writing

Though the relations between motivation and writing ability continue to maintain significance to writing researchers (Camacho et al., 2021a), relatively few studies have investigated the explicit profiles of students as writers. Those that have undertaken profiling have done so based on a variety of measures including ability and motivation—the former being the most prevalent in relative terms. The early work of Roid (1994) utilized cluster analysis to identify 11 unique patterns of student writing across various analytic domains, though inconsistencies existed within clusters. Later work saw the qualitative characterization of clusters of writers as “high/expert” or “low/poor” based on stable performance in domains ranging from spelling, grammar, and semantics (Wakely et al., 2006) to problem-solving, attention, self-monitoring, and language (Hooper et al., 2006). Such cognitive and linguistic measures aptly constitute the ability of a student writer. More recently, Coker et al. (2018) found that discrete profiles of writers based on similar metrics emerge as early as first grade. Of the five profiles derived by their latent profile analysis (LPA), students identified as “At Risk” consistently scored lower on factors related to quality/length, spelling, mechanics, and syntax. Similar profiles have even been identified among preschool children along related dimensions (Guo et al., 2018). Yet, as Coker et al. (2018) point out, measures of ability (often via assessments alone) fail to capture all the factors that influence writing success.

Accordingly, researchers have also explored student-level differences in motivation and its subsequent impact on writing outcomes, though these efforts have largely utilized methodologies that do not explicitly profile (e.g., MANOVA). For example, Troia et al. (2012) arguably approximated potential profiles of writers’ motivation, activity, and writing ability with consideration for the moderating effects of grade-level, sex, and ability for students in Grades 4 through 10. They found that motivation as measured by a beliefs survey showed a significant positive effect on narrative quality.

Troia et al. (2022) followed their prior research with a comprehensive investigation that explicitly profiled students in Grades 4 and 5 using LPA with various, interrelated dimensions of writing including ability, cognitive processes, motivation, and affect. Measures of writing ability included transcription fluency, vocabulary, spelling, mechanics, as well as general essay planning and quality. Cognitive measures included measures of discourse knowledge, working memory and word-reading skill because reading is fundamental for text interpretation and influences text length and quality. Finally, writing motivation was measured with the *Situated Writing Activity and Motivation Scale*, which directly addresses explicit aspects of motivation and both self-efficacy and outcome expectations for skills and tasks. The authors’ five-profile model suggested that in addition to the globally weak and globally proficient writers found in prior ability-focused research, there existed average-ability writers who varied significantly from each other on levels of motivation, perhaps moderating differential writing proficiency to some extent. Interestingly, globally proficient writers were nearly identical to both motivated and unmotivated average writers in most regards (e.g., component skills, working memory), except that the ability to demonstrate essay planning was uniquely sophisticated for only globally proficient writers.

De Smedt et al. (2022) also aimed at identifying writer profiles of Belgian high-school students using dimensions of writing that go beyond writing ability. Using a hierarchical cluster analysis, the authors identified two distinct clusters based on a scale measuring autonomous motivation (e.g., writing for enjoyment), internally imposed writing motives (e.g., writing to avoid the guilt of not writing), and externally imposed motives (e.g., writing to get a reward from a teacher), and a measure of students’ writing process. One of the identified clusters included process-oriented students with high autonomous motivation, whereas the second cluster included students that were less process-oriented and with less autonomous motivation. Similarly, Van Steendam et al. (2022) profiled Dutch high-school students based on their process configurations when completing source-based writing tasks. However, they did not include measures of motivation in their profiles.

Ng et al. (2022)’s clustering strategy exclusively used writing motives as the clustering variables. The authors found seven distinct clusters of Chinese fourth-grade students that differed on the extent to which they were motivated by curiosity, involvement, grades, competition, emotion, boredom, or social recognition. Clusters ranged from extremely motivated writers with high scores across all seven motives, to unmotivated writers with low scores across all motives. Other clusters had varying degrees of motives such as some students were focused on performance while others were predominately motivated by curiosity and involvement. This study used a strong combination of motives to cluster students, but it did not examine writing outcomes as part of the models.

Hence, further efforts to profile writers based on motivation and ability as they relate to writing are warranted, especially given motivation’s notable—and arguably understudied—role in the writing process (Boscolo and Gelati, 2019) and its complex relationship with writing proficiency (Ekholm et al., 2018). Moreover, recent contributions to profiling focused exclusively on students in upper elementary grades (Ng et al., 2022; Troia et al., 2022) or high school students (De Smedt et al., 2022; Van Steendam et al., 2022). Thus, there exists no research on how student writing ability/motivation profiles may differ at the middle-school level.

#### 1.1.3. Automated writing evaluation

AWE is intended to help students learn to write by accelerating the practice-feedback cycle (Kellogg et al., 2010) and supporting the cognitive and affective processes undergirding writing development. AWE feedback can range from basic (e.g., right or wrong answers) to highly complex, rich and individualized suggestions to improve writing (Fu et al., 2022). For example, AWE can provide detailed feedback on high-level traits (e.g., organization, development of ideas or style) alongside direct corrections of grammar and spelling mistakes, and suggestions for further learning such as video lessons teaching specific aspects of writing (e.g., Wilson and Roscoe, 2020). Other examples of more elaborate AWE feedback include explaining why an answer is right or wrong, or providing hints to guide students in their revisions (see a complete list in Fu et al., 2022).

AWE feedback is usually provided to students in and by the AWE software. However, learner-teacher interaction features allow for communication between students and teachers, and for teachers’ feedback to supplement the automated feedback (e.g., Wilson and Czik, 2016; Link et al., 2022). Several studies have explored the differential effects of teacher-, peer-, and computer-generated feedback (see Fu et al., 2022 for a systematic review on the topic). Although findings indicate significant positive effects of each feedback modality, teacher feedback generally has a stronger effect: Graham et al. (2015) report an average weighted effect size of 0.87 for teacher feedback compared to 0.38 for computer feedback. However, taken together, findings in this area suggest that blended feedback from AWE and a teacher or peer can lead to better writing outcomes (Fu et al., 2022).

By providing students with immediate feedback, students learn writing ability criteria. Knowledge of this evaluation criteria is fundamental to students being able to identify areas of improvement when reviewing their writing, and to revise their writing productively (MacArthur, 2016). Increased knowledge of evaluation criteria also may have benefits to students in terms of their confidence as writers (i.e., their self-efficacy). For instance, exposure to AWE feedback is associated with improvements in middle school students’ ability to accurately evaluate their writing ability (i.e., their calibration accuracy) and their self-efficacy for self-regulating the writing process (Wilson et al., 2022). Indeed, a quasi-experimental study found that middle schoolers using AWE to compose multiple essays had significantly greater self-efficacy for writing at follow-up compared to students using GoogleDocs after controlling for baseline self-efficacy (Wilson and Roscoe, 2020).

The immediacy of AWE feedback, as well as its potential for gamifying the writing process, may support improvements in students’ writing motivation, too. Several studies have found that elementary, middle, and secondary students report being more motivated to draft and revise their writing when using AWE (Warschauer and Grimes, 2008; Grimes and Warschauer, 2010; Ware, 2014; Moore and MacArthur, 2016; Wilson et al., 2021b). Indeed, evidence from a quasi-experimental study revealed that students using AWE self-reported significantly greater persistence for solving problems in their writing than students using GoogleDocs to compose. However, despite the general positive trend, several studies have reported negative associations between AWE feedback and writing motivation. For example, students may feel overburdened by the amount of feedback, perceive AWE feedback as less trustworthy than their teachers’ feedback, or feel discouraged when they receive vague feedback or low scores (see Wilson et al., 2021a; Fu et al., 2022).

With respect to improving students’ writing ability, several syntheses and meta-analyses indicate that AWE may be an effective writing intervention (Stevenson and Phakiti, 2014; Graham et al., 2015; Fu et al., 2022; Li, 2022). For instance, Graham et al. (2015) reported an average weighted effect size of 0.38 on writing ability for four studies of computer-based feedback. Li (2022) reported an overall effect (*g*) of 0.43 of AWE on writing ability for 25 studies published between 2000 and 2022. However, as with findings on motivation, there are exceptions to the trend of positive effects of AWE on writing outcomes. Individual differences in students’ literacy and language skills, as well as their motivation and attitudes toward writing, may moderate the effects of AWE on writing outcomes (Fu et al., 2022). Thus, the extent to which adolescents with different writing motivation/ability profiles respond uniformly to an AWE intervention remains to be seen.

### 1.2. Present study

Students exhibit heterogeneity in writing motivation and ability. Prior research has shown that this heterogeneity can be characterized into distinct profiles. However, prior research has often profiled writers based on measures of ability alone (Coker et al., 2018). Rarely have researchers profiled writers based on measures of both motivation and ability (c.f., Troia et al., 2022), yet such profiles might better describe the heterogeneity in students’ writing development. Further, such profiles might assist in better understanding the effects of promising technology-based writing interventions like AWE that are aimed at improving students’ writing outcomes, as students with different writing motivation and ability profiles may respond differently to an AWE intervention.

The present study addresses this gap through a randomized control experiment in which a sample of middle schoolers who were predominantly Black or Hispanic/Latinx and received FRL were randomly assigned to a business-as-usual English language arts (ELA) comparison condition or to an intervention condition in which they had access to the AWE system *MI Write* during their ELA instruction. We focus on this population because they are often overrepresented as struggling and low-performing writers (National Center for Education Statistics, 2012). We aim to answer the following research questions:

1. What are the writing motivation and ability profiles of diverse middle school students?
2. Are the identified profiles invariant across a school year and across different demographic groups?
3. What are the within-person and within-sample transition paths between these profiles across a school year, and what is the effect of an AWE intervention on these transitions?
4. Are there differences in students’ writing motivation and ability profiles and transition paths according to demographic predictors?

## 2. Materials and methods

### 2.1. Participants

We collected data from 2,487 students in Grades 7 and 8 (51.9% female) who were taught by 37 teachers participating in the randomized controlled trial. Three school districts in the Mid-Atlantic and Southern U.S. were invited to participate in the RCT because 50% or more of their student population was considered a *priority population*^1^, as defined at the time by the funding agency of this project (i.e., students were Hispanic/Latinx or Black and/or experiencing poverty as indicated by receiving FRL).

All seventh and eighth grade teachers across the 14 schools were invited to participate in the study and only two teachers opted out of participating after consenting (5.1% attrition, which is considered low by What Works Clearinghouse, 2017). Most students in the sample were in the eighth grade (68.4%). Students receiving special education comprised 6.2% of the sample. The sample included very few English-learners (ELs; 2.6%), as the school districts typically did not include ELs in their general education ELA courses. Table 1 displays participant demographics.

**Table 1:** Participant demographic information by intervention group.

	Comparison (BAU)		Intervention (MI)		Overall	
	*n*	%	*n*	%	*n*	%
Grade 8	715	58.3	986	78.3	1,701	68.4
Female	648	52.8	643	51.0	1,291	51.9
Priority population	951	77.5	1,027	81.5	1,978	79.5
Special education	76	6.2	109	8.7	185	7.4
English language learner	32	2.6	44	3.5	76	3.1
Total	1,227	49.3	1,260	50.7	2,487	100

*n*, number of participants; %, percentage of participants in group.

Intervention and comparison group subsamples did not differ with respect to gender (*χ*^2^_(1)_ = 0.79, *p* = 0.374) or EL status (*χ*^2^_(1)_ = 1.64, *p* = 0.200). However, the treatment group included a significantly higher proportion of students in the priority population (*χ*^2^_(1)_ = 6.12, *p* = 0.013, +4% difference) and students who received special education services (*χ*^2^_(1)_ = 5.45, *p* = 0.020, +3% difference).

Pretest equivalence on the writing motivation and writing ability measures was examined using independent sample *t*-tests. At pretest, students in the comparison and intervention groups did not differ in their self-efficacy for conventions (*p* = 0.055, *d* = 0.08), idea generation (*p* = 0.062, *d* = 0.08), or self-regulation (*p* = 0.076, *d* = 0.07). Likewise, there were no significant group differences in liking writing (*p* = 0.276, *d* = 0.04) or writing ability scores (*p* = 0.324, *d* = −0.04).

### 2.2. Design

We employed a randomized control trial with two data collection time points: the beginning and the end of the school year of 2021–2022. Randomization was performed at the teacher level using random number generation. To account for the nested structure of the data (i.e., students nested within teachers, within schools, within districts), we blocked teachers at the district, school, and grade level. This ensured that all teachers in all schools had an equal probability of receiving the intervention. Blocks of teachers were then randomly assigned to either a treatment (AWE intervention using MI Write) or comparison (business as usual ELA instruction) group. The research project had IRB approval. A total of 19 teachers were randomly assigned to the intervention group; 18 teachers were randomly assigned to the comparison group.

### 2.3. MI write

MI Write^2^ is an AWE system developed and marketed by Measurement Incorporated. It is designed to address the feedback burden on teachers, thereby allowing them to assign more writing and provide high-level feedback while allowing students to experience greater opportunities for writing practice and an accelerated practice-feedback cycle. This commercial tool is designed to be used by teachers and students in Grades 3–12 and provides a wide variety of features that support each agent in the writing process. MI Write uses an automated scoring engine, *Project Essay Grade* (PEG) to measure hundreds of linguistic indicators of writing ability that are used within a neural network to reliably predict human-assigned six trait scores (see Wilson et al., 2021b). Furthermore, PEG scoring produces specific grades and feedback depending on users’ grade-band (Grades 3–4, 5–6, 7–8, 9–10, 11–12), and task genre (informational, narrative, persuasive/argumentative).

MI Write offers electronic graphic organizers, interactive lessons, system and custom writing prompts, peer review, and multiple revision opportunities to support students’ deliberate writing practice (Palermo and Wilson, 2020). Secondly, MI Write’s automated scoring engine, *Project Essay Grade* (PEG) provides students with quantitative and qualitative feedback to help them calibrate their performance and revise and improve their writing. Quantitative feedback comes in the form of scores for six traits of writing. Qualitative feedback associated with each of the six traits of writing comes in the form of meta-cognitive prompts (e.g., *Does your writing have a clear conclusion?*) and suggestions for improvement (e.g., *Although your story is well developed, think about whether you can add even more details to improve your story*).

In addition, MI Write provides immediate, text-embedded grammar and spelling feedback, enabling students to make necessary edits to their essays. Teachers also may supplement MI Write’s feedback with summary comments and text-embedded in-line comments within their students’ writing. Findings from prior research indicate that MI Write has promise for improving students’ writing ability (Palermo and Thomson, 2018; Palermo and Wilson, 2020), self-efficacy and motivation to write (Wilson and Czik, 2016; Wilson and Roscoe, 2020; Wilson et al., 2022), and state test ELA performance (Wilson and Roscoe, 2020).

### 2.4. Measures

#### 2.4.1. Writing motivation and beliefs survey

The writing motivation and beliefs survey included two scales. First, students completed the Self-Efficacy for Writing Scale (SEWS) (Bruning et al., 2013), where they rated their confidence level to complete 19 writing tasks on a scale from 0 (*Not confident at all*) to 100 (*Completely confident*). Items were divided into three subscales: Conventions (five items; e.g., “*I can spell my words correctly*”), Idea Generation (six items; e.g., “*I can put my ideas into writing*”), and Self-Regulation (eight items; e.g., “*I can use feedback to improve my writing*”). Reliability for all scales was high at both pretest (α_Conv_ = 0.88; α_Idea_ = 0.92; α_SelfReg_ = 0.91) and posttest (α_Conv_ = 0.88; α_Idea_ = 0.93; α_SelfReg_ = 0.91).

Second, students reported their level of agreement with four statements about liking writing in the Liking Writing Scale (LWS; Bruning et al., 2013). Ratings ranged from 0 (*Strongly Disagree*) to 3 (*Strongly Agree*). Participants answered items such as “*I usually enjoy writing*,” and reverse-coded items such as “*I do not like to write*.” Higher scores in the LWS indicate higher liking of writing. This scale had good reliability at pretest (α = 0.84) and posttest (α = 0.86).

#### 2.4.2. Writing ability

Students wrote an argumentative essay in response to a source-based writing prompt at pretest and posttest (see prompts and links to sources in the Supplementary material). The prompt asked students to argue for or against certain uses of technology in society, specifically the use of computer-guided robots in the workplace (pretest prompt topic) and the use of voice-activated assistants (posttest). Students were given up to 75 min to read the sources, take notes, and plan, draft, and review their essay before submitting their essay electronically via Qualtrics. This genre was chosen because of its relevance to academic writing (MacArthur et al., 2015) and college readiness (Ray et al., 2019). Moreover, argumentative or persuasive writing using sources was part of the middle school ELA curricula of all three participating school districts. Therefore, all students had some previous experience with this type of writing.

Students’ prompts were scored for writing ability by PEG. PEG scores students’ writing on six traits: development of ideas, organization, style, word choice, sentence fluency, and conventions (range = 1–5). PEG also produces an Overall Score (range = 6–30) which is formed as the sum of the six traits. We adopted the Overall Score as the measure of writing ability in the current study because the individual trait scores were highly correlated (range *r* = 0.94–0.99), limiting their utility to provide unique information in a profile analysis. The PEG scoring system has been deemed valid and reliable in previous studies (Shermis, 2014; Wilson et al., 2019, 2022). Moreover, the Overall Score had high internal reliability at pretest (*α* = 0.99) and posttest (*α* = 0.99).

However, since we were using the PEG Overall Score as the sole measure of writing ability in the current study, we additionally sought to establish its convergent validity with a separate, validated human-scored measure of students’ argumentative writing quality, specifically the Smarter Balanced argumentative performance-task rubric for Grades 6–8. Smarter Balanced refers to the name of a consortium of US states and territories that utilize the Smarter Balanced assessment for yearly accountability assessments aligned with the Common Core state standards. This rubric was selected by the funding agency for use in our study because an independent panel of assessment experts deemed it to have excellent construct coverage and evidence of reliability and validity for the grade-level and across demographic subgroups. The rubric assesses organization/purpose, evidence/elaboration, and conventions. Ten percent of the entire corpus of baseline and follow-up essays were double scored among a pool of 12 raters to establish inter-rater reliability of the human scoring, which was strong: 57% exact agreement, 95% adjacent agreement, and *r* = 0.77. The Smarter Balanced scores were highly correlated with the PEG scores at both pretest (*r* = 0.78) and posttest (*r* = 0.84). Thus, this evidence supports the convergent validity of the PEG scores, indicating that PEG scores were not only reliable, but they provided a valid inference regarding students’ writing ability.

Table 2 presents descriptive statistics on motivation and ability measures at both time points. All measures of self-efficacy and the measure of writing ability significantly increased for the larger sample between pretest and posttest. However, liking writing significantly decreased at posttest. Standardized mean differences are reported in Table 2.

**Table 2:** Descriptive statistics of outcomes of interest.

	Time point 1		Time point 2		Standardized mean difference
	*N*	*M* *(SD)*	*N*	*M* *(SD)*	*d*
SEWS-conventions	2,431	72.9 (20.9)	2,364	77.3 (19.2)	0.29^***^
SEWS-idea generation	2,431	60.5 (23.8)	2,364	65.1 (23.1)	0.22^***^
SEWS-self-regulation	2,431	64.8 (23.1)	2,364	68.5 (22.0)	0.19^***^
LWS	2,428	1.7 (0.7)	2,364	1.7 (0.7)	−0.10^***^
Writing ability	2,243	15.8 (4.8)	2,186	17.6 (5.0)	0.39^***^

*N*, number of participants; SEWS; Self-Efficacy for Writing Scale; LWS, Liking Writing Scale; *d*, Cohen’s d. ^***^p < 0.001.

### 2.5. Procedure

This study was conducted during the 2021–2022 school year, during which lingering effects of the COVID-19 pandemic were still evident. We recruited school districts whose student body included over 50% Black or Hispanic/Latinx or students receiving FRL (i.e., students within the priority population). In the summer of 2021, ELA teachers provided consent to participate and all students in their Grade 7 and 8 rosters were given the opportunity to opt out of the study.

Prior to fall 2021, all participating teachers were trained by the research team to apply the pretest evaluation in their class. The research team was available for assistance. No participating teachers nor students had prior experience using MI Write. Therefore, teachers in the intervention condition followed a professional development plan during the year of implementation that consisted of one 2-h initial training in MI Write, and three professional learning sessions and at least five monthly coaching sessions (each 45–60 min) throughout the school year with Measurement Incorporated staff.

In October 2021, teachers administered the pretest in two sessions. In Session 1 (45 min), students completed the Writing Motivation and Beliefs Survey in Qualtrics (15 min) and reviewed two source articles for the argumentative essay. In Session 2 (45 min), students drafted and revised the argumentative essay. One school district completed the survey in 1 day and completed the entire writing task in a single 90-min session the following day to accommodate their schedule. In May 2022, teachers administered the posttest evaluation following the same protocol.

Across the 8 months of the study, students were intended to complete a total of eight pre-writing activities (i.e., MI Write electronic graphic organizers) and eight essays, revise each essay at least twice, engage in eight MI Write interactive lessons, and participate in three peer reviews. Teachers were expected to assign all these activities to students, and to provide feedback at least once to all student assignments submitted January through May (i.e., five assignments). MI Write logs collected data on all the aforementioned usage indicators for each teacher, specifically, the number of graphic organizers, prompts, lessons, and peer reviews teachers assigned, as well as the number of student essays teachers annotated. These logs were analyzed as a measure of fidelity of implementation. Teachers reported challenges meeting the implementation expectations stemming from teacher and student absences and remote and hybrid instruction.

### 2.6. Data analysis

All statistical models described in this section were estimated using Mplus 8.8 (Muthén and Muthén, 2018). There was no missing demographic data and the rates of missing survey data (7.5%) and essay responses (13.6%) were low, with differential attrition across treatment and comparison groups falling in the “Low” range for all measures (1.3% for survey measures and 5.6% for the essay) based on What Works Clearinghouse v.4.0 standards (2017). Thus, we used full information maximum likelihood (FIML) estimation in all models to handle missing data. FIML produces valid and unbiased parameters when data are assumed missing at random and have a multivariate normal distribution (Raudenbush and Bryk, 2002; Collins and Lanza, 2010; Cham et al., 2017).

#### 2.6.1. LPA and profile invariance

To answer RQ1, we first estimated LPA models separately at each time point using scores from the three SEWS subscales, LWS, and PEG Overall Score (i.e., writing ability) as indicators of the latent profiles. We tested solutions ranging from 1 to 6 latent profiles, with increasingly complex model configurations of variance-covariance structures.^3^ The optimal number of profiles was assessed with the Bayesian Information Criterion (BIC) for which lower values indicate better fit. A limitation of this criterion is that, with large sample sizes such as ours, it is likely that the value will not reach a minimum (Marsh et al., 2009). Therefore, we examined the gains associated with each additional profile in an “elbow” plot of the BIC values (Morin et al., 2011). Our final decision regarding the optimal profile solution was guided by theoretical interpretability, as is best practice (Johnson, 2021).

To answer RQ2, we tested whether the optimal profile solution remained invariant across time points. First, we linked the optimal profile solutions from each time point in a longitudinal model. We then tested profile invariance by comparing increasingly restrictive models (Morin et al., 2016; Morin and Litalien, 2017): (1) *configural* invariance (equal number of profiles identified at each time point), (2) *structural* invariance (equal profile means over time), (3) *dispersion* invariance (equal profile variances over time), and (4) *distributional* invariance (equal class probabilities over time). We repeated this process to test profile invariance across intervention (treatment vs. comparison) and demographic groups (i.e., separate models for gender groups, priority population groups, and special education groups) by fitting *configural* invariance, *structural* invariance, *dispersion* invariance, and *distribution* invariance models—note we did not test for invariance of the profile solution across EL and non-EL groups because of the very low percentage of ELs in our sample. Model fit was compared using BIC indices (Nylund et al., 2007).

#### 2.6.2. Latent transition analyses and predictors

After establishing profile invariance, we addressed RQ3 by fitting a latent transition model to test transition probabilities across profiles over time. Furthermore, we investigated RQ4 through various multigroup LTA models (Muthén and Asparouhov, 2011; Morin and Litalien, 2017). We conducted multigroup analyses separately using four binary predictors: intervention group, gender, priority population, and special education status. Once profile invariance was ensured as described in Section 2.6.1, we compared an LTA model in which the transition probabilities were free to vary across groups with a model version in which these probabilities were constrained to be equivalent across groups. We determined that there was a significant effect of the predictor on latent transitions when the model with free transition probabilities had a lower BIC value (i.e., had a better fit) than the model in which the transition probabilities were constrained to be equal across groups.

## 3. Results

### 3.1. Latent profiles of writing motivation and ability and profile invariance

Table 3 presents correlations among profile indicator variables. Model fit indices from the LPAs at both time points are shown in Table 4. First, we explored the BIC indices of each profile solution within each type of variance–covariance structure (see Footnote 3 for definitions). As expected, BIC indices continuedly declined with the addition of profiles. Therefore, we explored declines in BIC values using elbow plots and preferred the final profile solution to produce a large gain in model fit (see plots in this project’s OSF repository). Results for each variance–covariance structure were similar and BIC values flattened around four profiles for all structure types. Next, we compared BIC values across variance–covariance structures. The profile-varying non-diagonal structure had the lowest BIC value; however, we do not expect covariances to differ across profiles and thus chose a more parsimonious structure with the second lowest BIC (Johnson, 2021; Bauer, 2022). The optimal model was a four-profile solution with a profile-varying diagonal type variance-covariance structure. In this type of structure, indicator variances are allowed to differ in each profile, but they are “not allowed to co-vary over and above their association as part of the same profile” (Johnson, 2021, p. 124). The optimal profile solution was the same across time points.

**Table 3:** Correlations among outcome variables across time.

	1	2	3	4	5	6	7	8	9	10
1. SEWS-C 1	–									
2. SEWS-C 2	0.69	–								
3. SEWS-IG 1	0.67	0.48	–							
4. SEWS-IG 2	0.48	0.68	0.61	–						
5. SEWS-SR 1	0.72	0.54	0.82	0.57	–					
6. SEWS-SR 2	0.52	0.71	0.54	0.81	0.62	–				
7. LWS 1	0.20	0.25	0.48	0.33	0.48	0.33	–			
8. LWS 2	0.23	0.30	0.36	0.45	0.34	0.47	0.58	–		
9. WQ 1	0.34	0.34	0.21	0.23	0.29	0.31	0.21	0.22	–	
10. WQ 2	0.27	0.31	0.19	0.23	0.24	0.31	0.16	0.20	0.58	–

*N* = 2,487. SEWS-C 1 and -C 2 refer to the SEWS-Conventions subscale measured at pretest (1) and posttest (2). SEWS-IG 1 and -IG 2 refer to the SEWS-Idea generation subscale measured at pretest (1) and posttest (2). SEWS-SR 1 and -SR 2 refer to the SEWS-Self-regulation subscale measured at pretest (1) and posttest (2). LWS 1 and 2 refer to the Liking Writing subscale measured at pretest (1) and posttest (2). WQ 1 and WQ 2 refer to the writing ability scores measured by the PEG Overall Score at pretest (1) and posttest (2). All correlations are statistically significant at *p* < 0.001.

**Table 4:** BIC indices from LPAs of differing profile solutions and variance–covariance structures at each time point.

Profile solution	Type 1: profile invariant diagonal	Type 2: profile varying diagonal	Type 3: profile invariant non-diagonal	Type 4: profile varying non-diagonal
Time point 1 (pretest)				
1-profile	84582.34	84582.34	78987.93	78987.93
2-profile	80698.87	80150.44	78605.66	77942.05
3-profile	79478.07	78803.92	78450.16	77745.28
4-profile	79064.89	**78181.42**	78350.93	77736.94
5-profile	78881.22	78011.14	78301.00	77764.65
6-profile	78762.55	77907.54	78283.41	77812.60
Time point 2 (posttest)				
1-profile	81767.8	81767.8	76580.91	76580.91
2-profile	78017.62	77200.75	76064.98	75260.99
3-profile	76839.22	75838.01	75878.1	74996.31
4-profile	76445.95	**75243.66**	75740.26	74941.48
5-profile	76213.69	75050.78	75661.93	74906.18
6-profile	76117.16	74891.08	75613.89	–

The best-fitting solution (i.e., with the lowest BIC value) for each time point is in bold.

**Figure 1:**
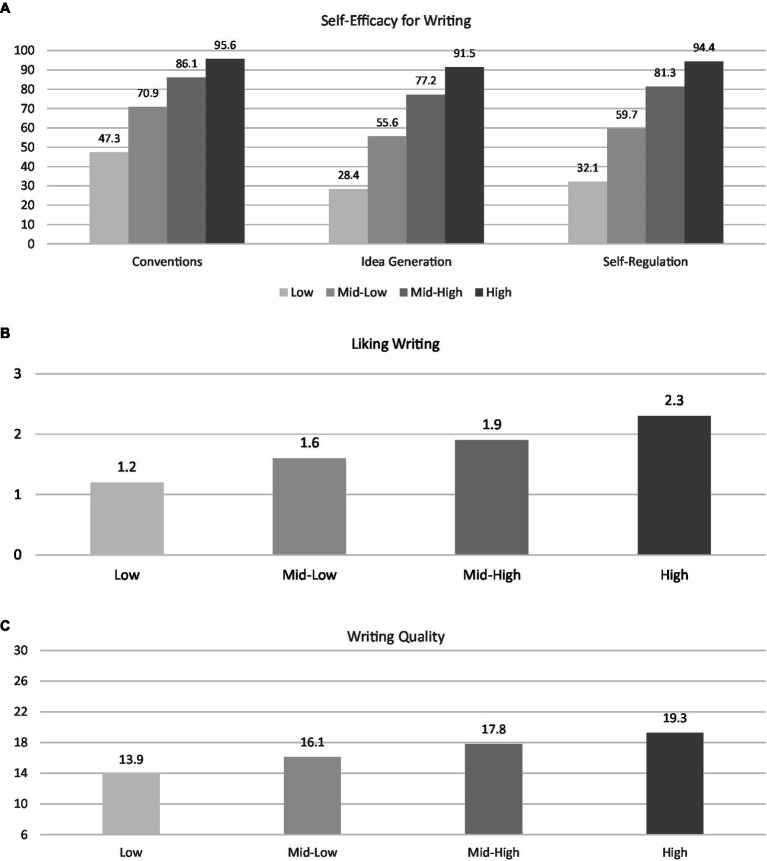
Profile means from the estimated model on **(A)** self-efficacy in writing, **(B)** liking writing, and **(C)** writing ability. *N* = 2,487. Because of the differences in ranges and to facilitate interpretation, means are plotted separately for each scale. Ranges: self-efficacy for writing subscales (0–100), liking writing (0–3), writing ability as measured by the PEG overall score (6–30).

Figure 1 displays the latent profile means and variances for the optimal model. Students in the *Low-Motivation and Ability (L-MA)* profile had the lowest scores on all indicators at both waves; means in this profile were well below the median for each indicator (e.g., a mean of 26 in self-efficacy for idea generation out of a possible score of 100). Next, students in the *Low/Mid-Motivation and Ability (LM-MA)* profile had slightly higher scores than the L-MA profile for all indicators at both waves. A *Mid/High-Motivation and Ability (MH-MA)* profile included students whose motivation and ability scores were higher than the previous profiles, and also higher than the median score for each indicator. Finally, a *High-Motivation and Ability (H-MA)* profile included students with scores near ceiling for self-efficacy indicators, and the highest scores on the LWS and in writing ability. Interestingly, students in the H-MA profile had writing ability scores only slightly above the median (i.e., 18 points within a range of 6–30).

Overall, most students started the school year in the LM-MA (38%) or MH-MA profiles (30.4%). The more extreme motivation and ability profiles included about a third of the sample, with students being more likely to start the school year in the L-MA profile (20.3%) than in the H-MA profile (11.3%). This distribution of students across profiles remained stable at the end of the school year, as indicated by the results of the invariance testing described next.

Indeed, we evaluated profile invariance across time points, treatment, and demographic groups by following the procedure described in Section 2.6.1. Table 5 shows BIC values for all profile invariance testing models. Changes in model fit as indicated by declining BIC values supported *distributional* invariance across time points. This suggests that the number of identified profiles, the profile means and variances, and the class probabilities (i.e., class sizes) remained stable across time points. Furthermore, invariance testing across treatment and demographic groups supported *dispersion* invariance, meaning the number of profiles, and profile means and variances were the same across treatment, gender, priority population, and special education groups. Although distributional invariance was not supported for demographic comparisons, that level of invariance was not desirable for our group invariance testing because the equality of class sizes is a highly restrictive assumption of little interest for researchers (Talley, 2020).

**Table 5:** BIC values for measurement invariance tests across time and demographic groups.

Predictor	Groups	Configural	Structural	Dispersion	Distributional
Time	Time 1 (pretest)	81865.85	81730.23	81590.70	**81571.30**
	Time 2 (posttest)	78776.82	78628.19	78554.24	**78539.48**
Intervention	Comparison	75534.66	75552.56	**75441.14**	75449.06
	Treatment	77101.87	77036.78	**76923.27**	76954.03
Gender	Male	73327.71	73312.15	**73196.93**	73216.27
	Female	78941.36	78914.70	**78792.69**	78807.83
Priority population	Non-priority	31459.11	31383.46	**31282.44**	31278.62
	Priority	120998.36	121051.11	**120934.29**	120972.87
Special education	General education	140167.01	140223.53	**140106.86**	140145.57
	Special education	11936.13	11872.16	**11829.68**	11822.39

The best-fitting solution (i.e., with the lowest BIC value) for each time point is in bold.

### 3.2. Latent transitions and predictors

The tests of transition probabilities by group showed no significant differences by assignment to intervention, gender, priority population status, or special education status. For each predictor, the model where transition probabilities were constrained to be equal across groups had lower BIC values and, therefore, fit better than a model with freely estimated transition probabilities (see Table 6). Given that the chosen predictors did not yield differences in transition probabilities, the transition probabilities described in this section approximately describe all students irrespective of their assignment to intervention, gender, priority population status, or special education status (see Table 7).

**Table 6:** Fit statistics of models comparing whether transition probabilities differed across groups.

	Free transition probabilities	Equal transition probabilities
Intervention group	155627.84	**155572.39**
Gender	155616.14	**155569.19**
Priority population	154692.03	**154645.94**
Special education status	153485.38	**153442.43**

The best-fitting solution (i.e., with the lowest BIC value) for each time point is in bold.

**Table 7:** Estimated latent transition probabilities across time points.

	Time 2 profile			
Time 1 profile	L-MA	LM-MA	MH-MA	H-MA
L-MA	0.549	0.390	0.059	0.002
LM-MA	0.085	0.568	0.318	0.029
MH-MA	0.018	0.163	0.597	0.223
H-MA	0.003	0.029	0.263	0.705

L-MA, Low-Motivation and Ability; LM-MA, Low/Mid-Motivation and Ability; MH-MA, Mid/High-Motivation and Ability; H-MA, High-Motivation and Ability.

Overall, the most probable path was for students to remain in the motivation and ability profile where they started the school year. The most stable profile over time was the H-MA profile: 70.5% of students who started in this profile remained in it at the end of the school year. The other profiles were stable for approximately half of students (L-MA = 54.9%; LM-MA = 56.8%; MH-MA = 59.7%). For students in the L-MA, LM-MA, and MH-MA profiles, the next most probable transition was to move one profile higher. For example, approximately 30% of students in the L-MA profile moved to a LM-MA profile by the end of the school year.

The probabilities of students moving two or more profiles higher (e.g., from L-MA to MH-MA or to H-MA) were low (i.e., less than 6%) or extremely low (i.e., less than 1%), respectively. Approximately 26% of students in the H-MA profile dropped to the MH-MA profile, and only 16% of students in the MH-MA profile dropped to the LM-MA profile. Notably, the probabilities of students dropping to the L-MA profile were below 8% for all other profiles.

## 4. Discussion

The purpose of this investigation was to deepen the understanding of middle-school students’ writing motivation and ability by identifying distinct profiles that could characterize the relations between these constructs during middle school. We focused on traditionally struggling writers and implemented our profiling strategy with a majority of Black and Hispanic/Latinx students who received FRL (National Center for Education Statistics, 2012). Furthermore, we investigated the invariance of these profiles over a school year and across various demographic groups (i.e., gender, priority population status, and special education status). After establishing profile invariance, we explored the differences in transition paths among profiles across a school year as a result of being assigned to an AWE intervention, and potential effects of gender, priority population status, and special education status.

### 4.1. Motivation and ability profiles of middle school students

Our study is the first to profile students based on writing motivation and ability during middle school. Notably, results from the LPA at both time points indicated four distinct profiles of writing motivation and ability: Low-, Low/Mid-, Mid/High-, and High-Motivation and Ability profiles. Consistent with prior research, the measures of self-efficacy, writing attitudes, and writing ability used in the profiling were strongly aligned with one another (Bruning et al., 2013; MacArthur et al., 2016; Camacho et al., 2021b). In other words, students with the highest levels of self-efficacy also liked writing the most and achieved the highest writing quality relative to other students in the sample. Therefore, the four profiles differed in terms of the level of each construct, but not the pattern of relations between the constructs as can occur with latent profiling (Johnson, 2021).

The profiles identified in this paper suggest that writing self-efficacy, attitudes, and ability are positively related. Previous studies have explored how writing self-efficacy and attitudes contribute to writing quality (e.g., Graham et al., 2019; Wijekumar et al., 2019; Camacho et al., 2021b), but no research to date had explored the relations among these constructs in a latent profiling strategy that allows them to change together and allows these interactions to change among groups of students. Therefore, our findings contribute to the ongoing debate about the multiple and distinct constructs under the umbrella term of writing motivation, and how these relate to one another and to writing performance (Abdel Latif, 2019). Moreover, our finding that all constructs have stable relations supports the idea that writing self-efficacy and attitudes may be reasonable constructs to target when aiming to improve students’ writing performance. To elaborate on this finding, future research could include measures of other motivational constructs beyond self-efficacy and liking writing for building the profiles and ascertain whether motivation and attitudes remain as strongly linked within profiles as they were in the profiles identified in the present study. For example, it would be beneficial to use a comprehensive account of different writing motives, such as in the cluster analysis by Ng et al. (2022), that used the seven motives proposed by Graham et al. (2022): curiosity, involvement, grades, competition, emotion, boredom, or social recognition.

Our second research question assessed whether the identified profiles were applicable across demographic groups, but other studies using LPA have instead explored the effect of demographic variables on profile membership using students’ most likely profile in a logistic regression (e.g., Troia et al., 2022). Nonetheless, this analytic procedure does not account for the classification error of the latent probabilities of being assigned to the other profiles in the model and can, therefore, yield biased model estimates (Bakk and Kuha, 2020). Given that our study assessed a slightly different question, and that we wanted to account for the classification error when exploring how the profiles looked like with various demographic groups, we opted to do a profile invariance analysis with several multigroup models (Muthén and Asparouhov, 2011; Morin and Litalien, 2017).

Results from our second research question indicated that the four identified profiles apply similarly to different demographic subgroups including gender, priority population status, and special education status. Previous profiling efforts identified particular writing ability profiles for at-risk students (e.g., Coker et al., 2018); thus, we hypothesized that motivation and ability profiles might differ across demographic groups. Our findings disproving differences in profiles imply that writing motivation and ability profiles using self-efficacy and attitudes toward writing measures look similar for boys and girls, priority and non-priority students, and special education and general education students at the middle school level. Thus, for purposes of screening students, our profiling strategy appears to be feasible and valid for wide application.

Moreover, we found that almost 40% of students started the school year in the Low-Mid profile (38%) or the Low profile (20.3%), which is consistent with prior LPA research conducted by Troia et al. (2022) with elementary school students. Taken together, these results unfortunately confirm the rather discouraging levels of writing motivation and ability among US students. However, profiling students within comprehensive and multidimensional models of writing that include measures of motivation and ability, and even cognitive processes or other beliefs, allows researchers and practitioners to have a better understanding of the starting point to intervene and, eventually, improve students’ writing during in middle school.

### 4.2. Transition paths with and without predictors

To answer our third research question, we investigated the transition paths among profiles across a school year, first without including predictors (i.e., assignment to treatment and demographic predictors). Next, we included the predictors, but found that profiles were invariant and, thus, assignment to treatment and demographic characteristics did not influence how students transitioned across profiles in a school year.

Our finding that the most common path was for students to begin and end the school year in the same profile suggests students generally have stable writing motivation and ability within a school year. This transition path was especially prevalent for the H-MA students (70.5%), which is encouraging for students that start the year motivated and demonstrating strong writing abilities. However, these students are the minority: only 11.3% of students are in the H-MA profile at the beginning of the year.

Stability within profiles over the school year was slightly less common for students who began in the L-MA and L/M-MA profiles (54.9 and 56.8%, respectively). The next most probable path for these students was to improve slightly and move one profile up (approximately 30% of students transition in this path). While this suggests a trend of slight improvements, most students who start the year unmotivated and exhibiting weak writing skills retain these characteristics after a full year of instruction and additional aids (i.e., AWE intervention).

Taken together, these results present two challenges. First, the general stability of the high ability-motivation (H-MA) profile suggests that these students might lack room to grow in their motivation albeit they can improve in their writing ability. This highlights the need to design challenges to maintain students’ motivation and improve their writing ability. Second, the general stability of the lower profiles reinforces the importance of developing interventions to offset the typical course of action, that is, students remain in their profile or worsen over time (as they advance through middle school and high school; Wright et al., 2020).

One such intervention could be using technology-based tools, like AWE, that provide students with more feedback on their writing abilities, and actionable steps to improve them. We investigated the effects of an AWE intervention using MI Write on transition paths; unfortunately, being assigned to receive this intervention did not change these paths. One reason for this might be the fact that our study was done in the context of an RCT, and in this paper we specifically evaluated whether assignment to treatment was impactful on motivation, not if adherence to treatment had an impact. However, it is reasonable that there is likely a threshold of AWE usage that is required before impacts on motivation and ability profiles are manifested. Future research should seek to identify this threshold. Also, additional research should be conducted with other technology-based writing interventions, such as intelligent tutoring systems (e.g., Wijekumar et al., 2022), to identify whether results are idiosyncratic to AWE or whether the motivational effects associated with such other interventions (Morphy and Graham, 2012) yield similar findings.

Secondly, and importantly, the intervention in this study did not incorporate explicit methods of improving writing motivation and was aimed primarily at improving writing ability through the provision of frequent, immediate, and informative automated feedback. Previous studies have suggested that AWE can support motivation (e.g., Moore and MacArthur, 2016; Wilson and Roscoe, 2020), but AWE by itself does not directly address motivation constructs (e.g., by providing feedback about attitudes or beliefs about writing). In contrast, other types of interventions that deliberately target writing motivation have shown some degree of positive results on writing motivation, for example self-regulated strategy development interventions, strategy instruction combined with a process approach, collaborative writing, creative writing, linguistic games, drama theater interventions, or interventions where teachers deliberately adopt motivation-enhancing strategies (see Camacho et al., 2021a for a review). Indeed, explicitly incorporating a goal-setting intervention with AWE has shown promise for improving adolescents’ self-efficacy for self-regulation (Wilson et al., 2022). Our results prove that incidental motivational gains promised by AWE are not enough to create meaningful changes in motivational profiles. Hence, future intervention studies, especially those that focus on AWE, may benefit from adding components that specifically target writing motivation alongside components to improve writing ability.

### 4.3. Limitations and future directions

One limitation pertains to our participant sample and the demographic predictors used in the LPA. Participating schools in our study were exclusively those serving a high proportion of priority population students. While our findings based on this sample help to diversify current literature that has oversampled White, middle-to-high-income students, our priority vs. non-priority comparisons may not generalize to a different sample. Our findings are subject to similar limitations regarding special education status. Only 7% of our participants received special education services; therefore, a study with greater representation of students with disabilities would aid in understanding the motivation and ability profiles of these students.

The design employed as part of the present study has the strength of randomly assigning students to either an AWE intervention or to receive business-as-usual ELA instruction. Nonetheless, there were some limitations to consider when discussing our findings. First, the analyses in the present study focus on assignment to treatment and not necessarily on treatment itself. While we had specific usage guidelines and measures of fidelity of implementation, the limitations of teaching and collecting data during a global pandemic meant that some of the thresholds for fidelity were not met (see Wilson et al., 2023). Therefore, students in our sample received different dosages of the AWE intervention. Future studies should evaluate the impact of the intervention under different dosage conditions, as the nonsignificant effect of assignment to treatment found in this study might change when the dosage of treatment is considered. Results of our study should be interpreted akin to an intent-to-treat analysis (vs. a treatment-on-the-treated analysis), revealing the transition paths associated with *providing access to MI Write* but not necessarily indicating those paths that would be associated with different thresholds of MI Write usage.

Finally, the profiles of writing motivation and ability in our study are limited to a global measure of writing ability. This global measure was chosen to fit with the self-efficacy in writing and writing attitudes measures that asked students about their ideas about writing as a general process, and their skills as writers without specifying genres or processes. Previous studies have profiled students in writing ability using multiple detailed measures, for example spelling, grammar and semantics (Wakely et al., 2006); quality/length, spelling, mechanics, and syntax (Coker et al., 2018; Guo et al., 2018); and handwriting and typing fluency, punctuation, spelling, reading, vocabulary (Troia et al., 2022). Thus, future research on profiles of writing motivation and ability can be expanded to include detailed measures of writing ability, or even task- or genre-specific measures (see Troia et al., 2022) for a more comprehensive perspective on how motivation and ability relate in middle school students.

## Data availability statement

The datasets presented in this study can be found in online repositories. The names of the repository/repositories and accession number(s) can be found below: https://osf.io/fgb7k/?view_only=953f94eab8674e7b9bb6be84d139a7d9.

## Ethics statement

The studies involving human participants were reviewed and approved by Institutional Review Board University of Delaware. Written informed consent from the participants’ legal guardian/next of kin was not required to participate in this study in accordance with the national legislation and the institutional requirements.

## Author contributions

TC: conceptualization, methodology, writing - original draft, formal analysis, visualization, data curation. JW: conceptualization, methodology, writing - original draft, resources, supervision, funding acquisition. MM: writing - original draft, investigation. CP: software, resources, writing - review & editing, supervision, funding acquisition. HE and JC: project administration. AP: Investigation.

## Funding

This work was supported, in whole or in part, by the Bill & Melinda Gates Foundation [INV-006167]. The opinions expressed in this paper are those of the authors and do not represent the views of the Foundation, and no official endorsement by this agency should be inferred.

## Conflict of interest

CP, HE, and JC were employed by Measurement Incorporated.

The remaining authors declare that the research was conducted in the absence of any commercial or financial relationships that could be construed as a potential conflict of interest.

The handling editor SG declared a shared affiliation with the author(s) AP at the time of review.

## Publisher’s note

All claims expressed in this article are solely those of the authors and do not necessarily represent those of their affiliated organizations, or those of the publisher, the editors and the reviewers. Any product that may be evaluated in this article, or claim that may be made by its manufacturer, is not guaranteed or endorsed by the publisher.

## Footnotes

^1^This term has since been updated by the funding agency and is now termed “communities in focus.”

^2^ www.miwrite.com

^3^The variance-covariance structure types are described based on the definitions by Johnson (2021): Type 1 = indicator variances are equal across profiles, and covariances are constrained to zero; Type 2 = indicator variances are freely estimated across profiles, and covariances are constrained to zero; Type 3 = variances are equal across profiles and covariances are estimated and constrained to be equal across profiles; Type 4 = both variances and covariances are freely estimated across profiles.
