# Supplementary material for: Writing motivation and ability profiles and transition during a technology-based writing intervention
Source: Front Psychol. 2023 Jun 21;14:1196274. doi: 10.3389/fpsyg.2023.1196274 (PMC10321671; doi:10.3389/fpsyg.2023.1196274)
Supplement: Supplementary file 1 [file Data_Sheet_1.pdf]

Supplementary Material

Writing Motivation and Ability Profiles and Transition During a Technology-Based Writing Intervention

Tania Cruz Cordero*, Joshua Wilson*, Matthew C. Myers, Corey Palermo, Halley Eacker, Andrew Potter, Jessica Coles

*** Correspondence:** Corresponding Author: joshwils@udel.edu

1 Writing Instructions, Prompt and Sources at Pretest and Posttest

1.1 Pretest

Computer-Guided Robots

BACKGROUND:

In recent years, a heated debate has emerged about the safety, security, economic, and ethical considerations of computer-guided robots in the workplace. As technology develops, the skill-sets of these robots are growing and becoming more versatile. This makes it possible for automated machines to more easily, safely, and efficiently complete a growing number of tasks previously completed by human workers.

The controversies surrounding this topic are related to the ways computer-guided robots in the workplace impact humans. Will these types of robots cause more unemployment? Is our human workforce properly educated and trained to work well with this new technology?

As you begin to research this topic, you uncover two sources about the technological, ethical, economic, and safety implications of using computer-guided robots in the workplace.

Your English Language Arts (ELA) class is publishing a literary magazine to highlight student writing. The magazine will be read by students, teachers, and parents. Your initial research has inspired you to write an argumentative essay for the magazine that addresses these issues surrounding computer-guided robots in the workplace.

DIRECTIONS:

Use the research sources to write a multi-paragraph essay in which you argue either for or against the use of computer-guided robots in the workplace. Make sure you establish an argumentative claim, support your claim drawing from the sources you have read, and discuss competing or alternate arguments.

Develop your ideas clearly and use your own words, except when quoting directly from the sources. Be sure to reference the sources by title or number when using details or facts directly from the sources.

Manage your time carefully so that you can

- plan your multi-paragraph argumentative essay.
- write your multi-paragraph argumentative essay.
- revise and edit the final draft of your multi-paragraph argumentative essay.

Download and/or listen to the following articles:

Article 1: "How Might Robots Change the Workplace of the Future?"

Article 2: "Artificial Intelligence and the Workplace"

1.2 Posttest

Voice-Activated Assistants

BACKGROUND:

In recent years, voice-activated assistants such as Alexa and Siri have become increasingly popular due to the many ways they provide convenience and make life easier. As this technology evolves, it'll potentially provide even more benefits to its users, saving them time and allowing them to be more efficient.

But a few significant concerns have arisen about voice-activated devices, sparking a debate about whether this technology should be integrated too deeply into our lives.

The main concern about this technology is related to privacy and the responsibility of its creators in protecting the sensitive data it gathers. Among other things, there are also long-term concerns about the impact voice-activated assistants have on our social skills and attention spans.

Do the conveniences this technology provides really outweigh the privacy risks and other concerns?

As part of your initial research, you have uncovered two sources about the technological and ethical implications of voice-activated devices.

Your English Language Arts (ELA) class is publishing a literary magazine to highlight student writing. The magazine will be read by students, teachers, and parents. Your initial research has inspired you to write an argumentative essay that addresses issues surrounding voice-activated devices.

DIRECTIONS:

Use the research sources to write a multi-paragraph essay in which you argue either for or against the use of voice-activated devices in homes in the United States. Make sure you establish an argumentative claim, support your claim drawing from the sources you have read, and discuss competing or alternate arguments. Develop your ideas clearly and use your own words, except when

quoting directly from the sources. Be sure to reference the sources by title or number when using details or facts directly from the sources.

Manage your time carefully so that you can

- plan your multi-paragraph argumentative essay.
- write your multi-paragraph argumentative essay.
- revise and edit the final draft of your multi-paragraph argumentative essay.

TASK PROMPT:

After reading two sources about voice-activated assistants, write a multi-paragraph essay in which you argue either for or against the use of voice-activated devices in homes in the United States. Discuss competing or alternate arguments. Support your claim with evidence from the texts.

Download and/or listen to the following articles:

Article 1: "Shhh, Amazon's 'Alexa' could be listening to our conversation"

Article 2: "Smart Home, Smart Move? The Pros and Cons of Voice Assistants"
